# Supplementary material for: Genomic insight into domestication of rubber tree
Source: Nat Commun. 2023 Aug 2;14:4651. doi: 10.1038/s41467-023-40304-y (PMC10397287; doi:10.1038/s41467-023-40304-y)
Supplement: Supplementary file 1 — Supplementary Information [file 41467_2023_40304_MOESM1_ESM.pdf]

# Genomic insight into domestication of rubber tree

Chao *et al.*

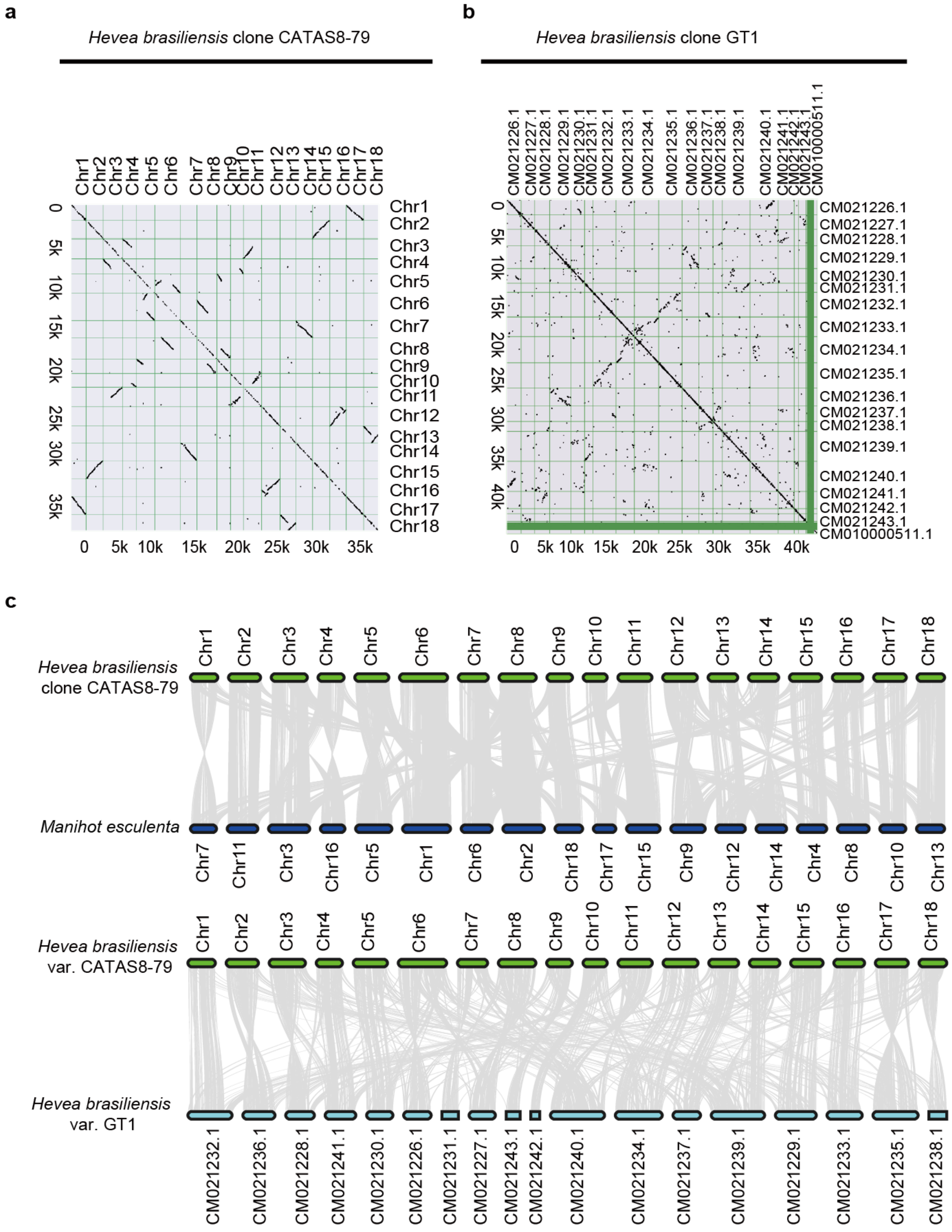


**Supplementary Figure 1. Synteny analysis.** (a) Intra-genome syntenic analysis of CATAS8-79 genome. (b) Intra-genome syntenic analysis of published GT1 genome. (c) Inter-genome syntenic analysis between CATAS8-79 and cassava, and between CATAS8-79 and GT1.

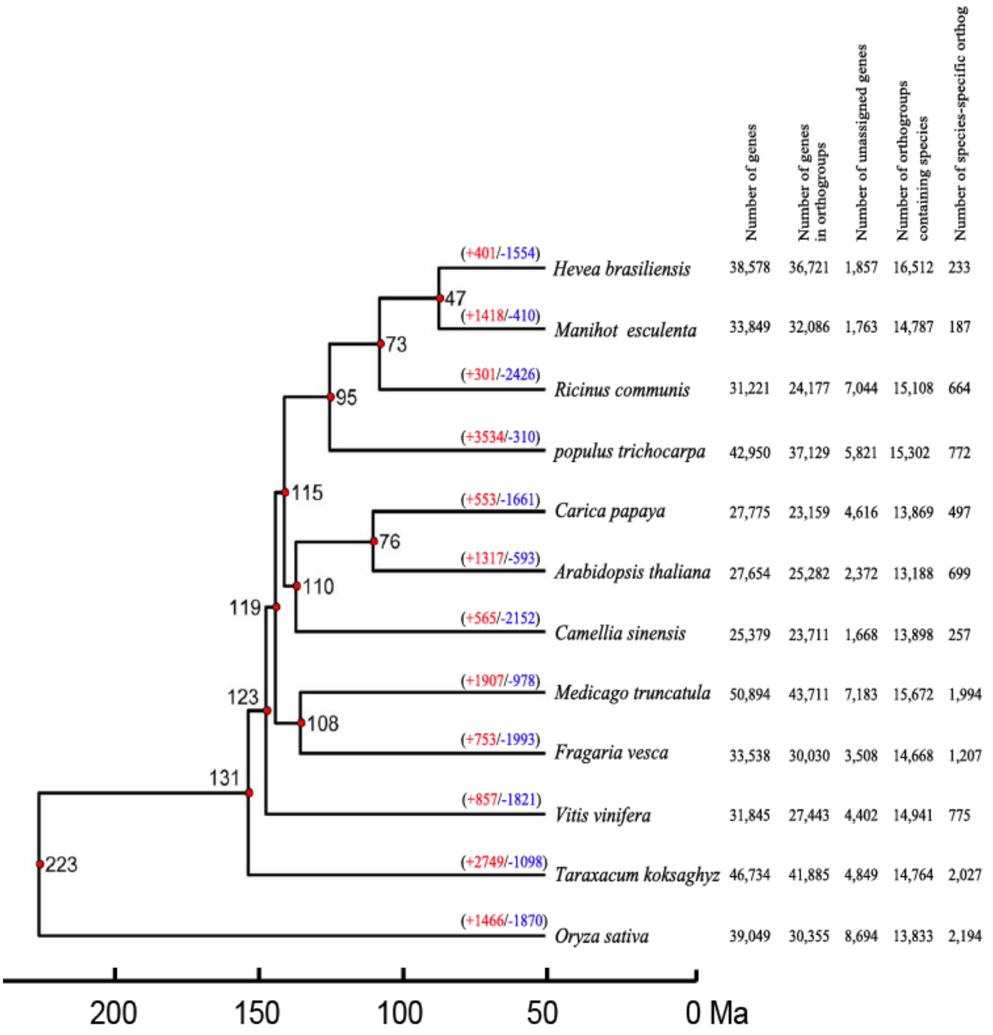


**Supplementary Figure 2. Analysis of gene family among 12 species.** Phylogenetic tree of 12 species including their divergence time with the aid of orthologues from single gene families. The number in each node indicated Ma between two divergent branches. Red/blue numbers indicated expansion/contraction of gene families. The five columns of numbers listed at the right of each species were counts of each feature of the gene families. *Oryza sativa* was used as an outgroup. Source data are provided as a Source Data file.

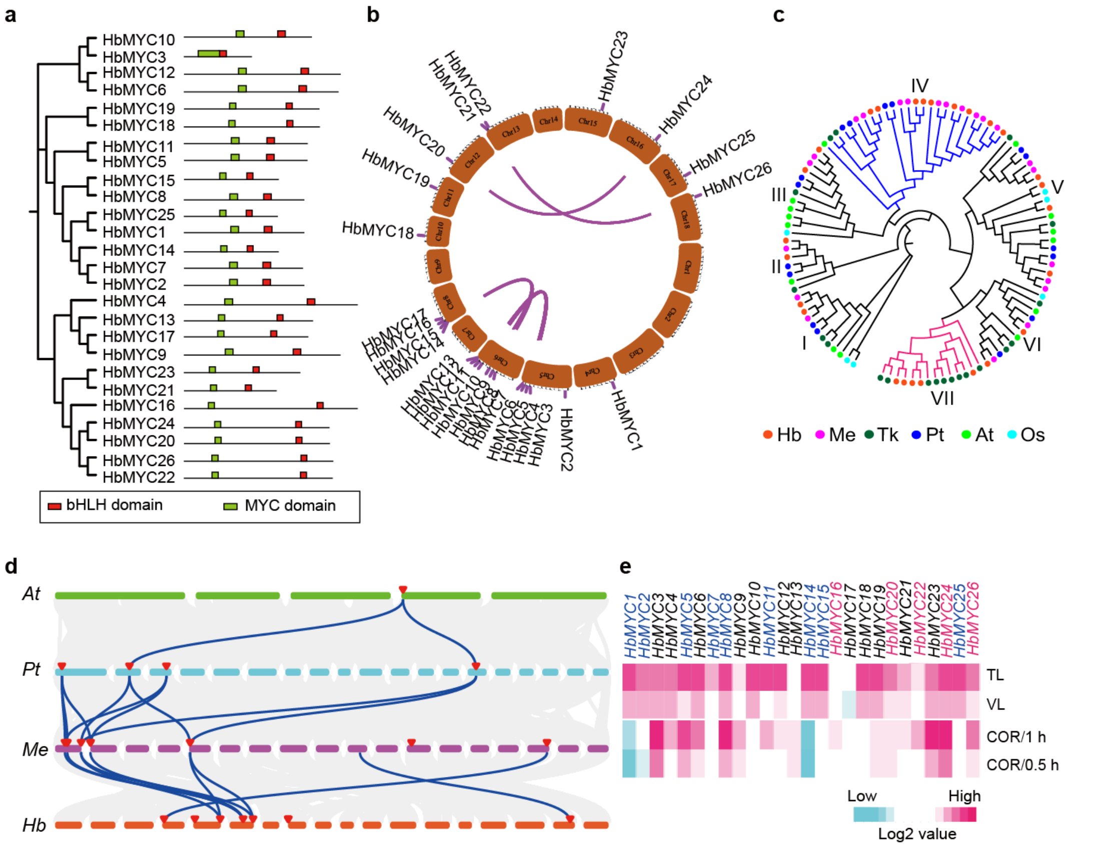


**Supplementary Figure 3. Identification of *HbMYC* gene family in CATAS8-79 genome.** (a) Motif analysis of *HbMYCs* using the MEME program. The red motif represented bHLH domain while the green motif represented MYC domain. (b) Location and interchromosomal relationships among *HbMYCs* in CATAS8-79 genome. Coloured lines indicated syntenic blocks. (c) Phylogenetic tree of MYC proteins. I-VII shows seven clades. At: *Arabidopsis thaliana*; Os: *Oryza sativa*; Pt: *Populus trichocarpa*; Tk: *Taraxacum kok-saghyz*; Me: *Manihot esculenta*; Hb: *Hevea brasiliensis*. (d) Synteny analysis of four species showing the expansion of MYC members. (e) Expression patterns of 26 *HbMYCs* in the latex from virgin trees (VL) and tapped trees (TL), and in the cambial region upon COR treatment for 0.5 h (COR/0.5 h) and 1 h (COR/1 h). The bar indicated the relative expression levels by color. Source data are provided as a Source Data file.

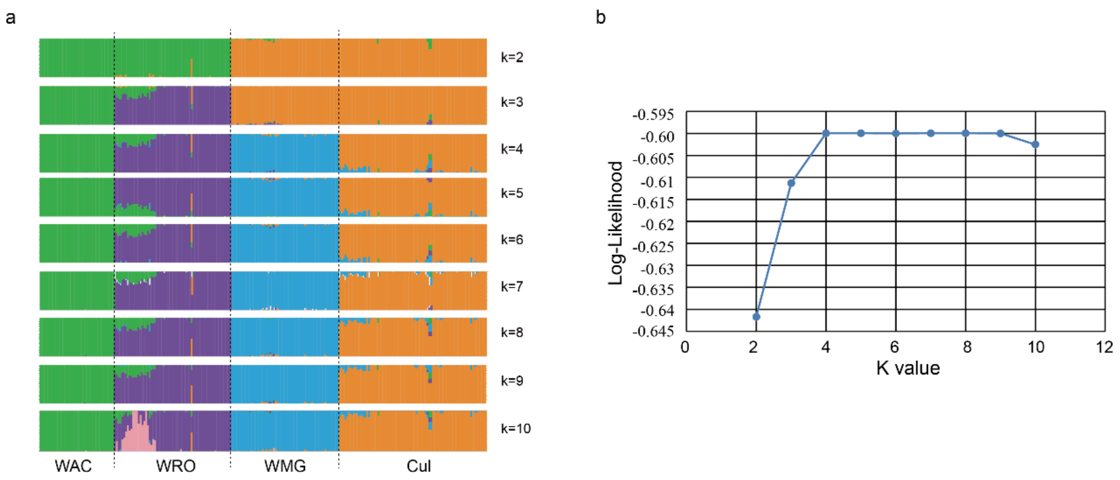


**Supplementary Figure 4. Population structure analysis. (a)** Population structure analysis of 245 individuals after removing the cultivars with rubber tree primary clone Tjir1' or PR107' pedigree and the admixed individuals based on phylogenetic analysis. (b) K value determination by Log-Likelihood curve generated by fastStructure software. Source data are provided as a Source Data file.

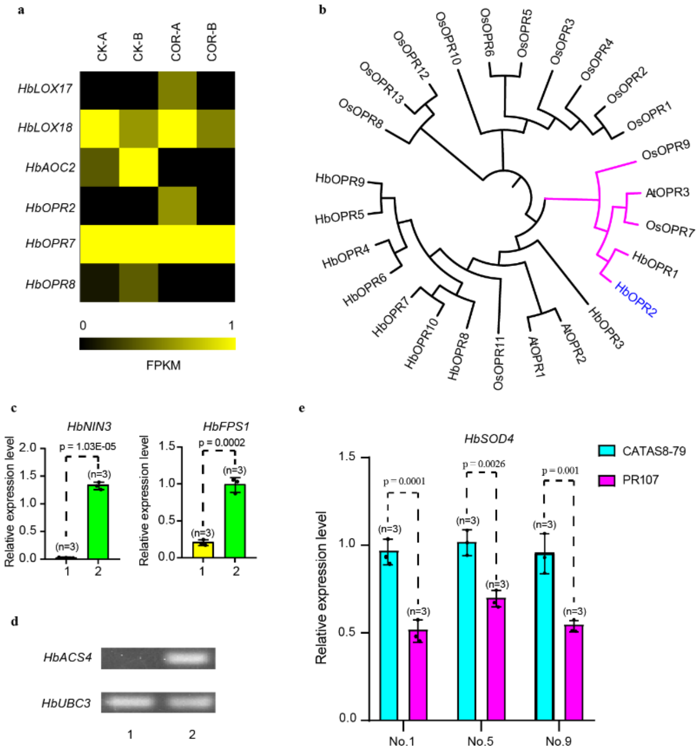


**Supplementary Figure 5. Relationship between rubber productivity and differential expression patterns of genes detected in selective sweeps.** (a) Relationship between rubber productivity and differential expression patterns of JA biosynthesis-related genes in the inner bark tissues of epicormic shoots of rubber tree clone CATAS7-33-97 treated with coronatine (COR), a mimic of active JA, considering endogenous JA was crucial to laticifer formation. The heatmap was based on transcriptome data deposited into NCBI (SRR3423347, SRR3423348, SRR3423349, and SRR3423350). The bar indicated FPKM values. (b) Phylogenetic tree of OPR proteins from rubber tree, rice and *Arabidopsis*. (c) Relationship between rubber productivity and differential expression patterns of *HbNIN3* and *HbFPS1* in latex from virgin trees (1) and regularly-tapped trees (2) of rubber tree clone CATAS7-33-97, considering the latex regeneration and rubber biosynthesis were remarkably activated in regularly-tapped trees in comparison to the virgin trees. *Hb18S* was used as reference gene. The results are expressed as means ± s.d. in three biological replicates. *P* values were calculated with two-sided Student's *t*-test. ***$P < 0.001$; ****$P < 0.0001$. (d) Relationship between rubber productivity and differential expression pattern of *HbACS4* in the inner bark tissue of trunks of virgin trees (1) and regularly-tapped trees (2) of rubber tree clone CATAS7-33-97, considering exogenous ethylene was effective in prolonging duration of latex flow and the duration of latex flow was much longer in regularly-tapped trees than that in virgin trees. The results were repeated two times. *HbUBC3* was used as reference gene. (e) Relationship between rubber productivity and differential expression pattern of *HbSOD4* in latex collected from the virgin trees of rubber tree clones CATAS8-79 and PR107 at the first (No.1), fifth (No.5) and ninth (No.9) tapping, considering the duration of latex flow was much longer in rubber tree clone CATAS8-79 than that in PR107. *HbUBC2b* was used as reference gene. The results are expressed as means ± s.d. in three biological replicates. *P* values were calculated with two-sided Student's *t*-test. **$P < 0.01$; ***$P < 0.001$; ****$P < 0.0001$. Source data are provided as a Source Data file.

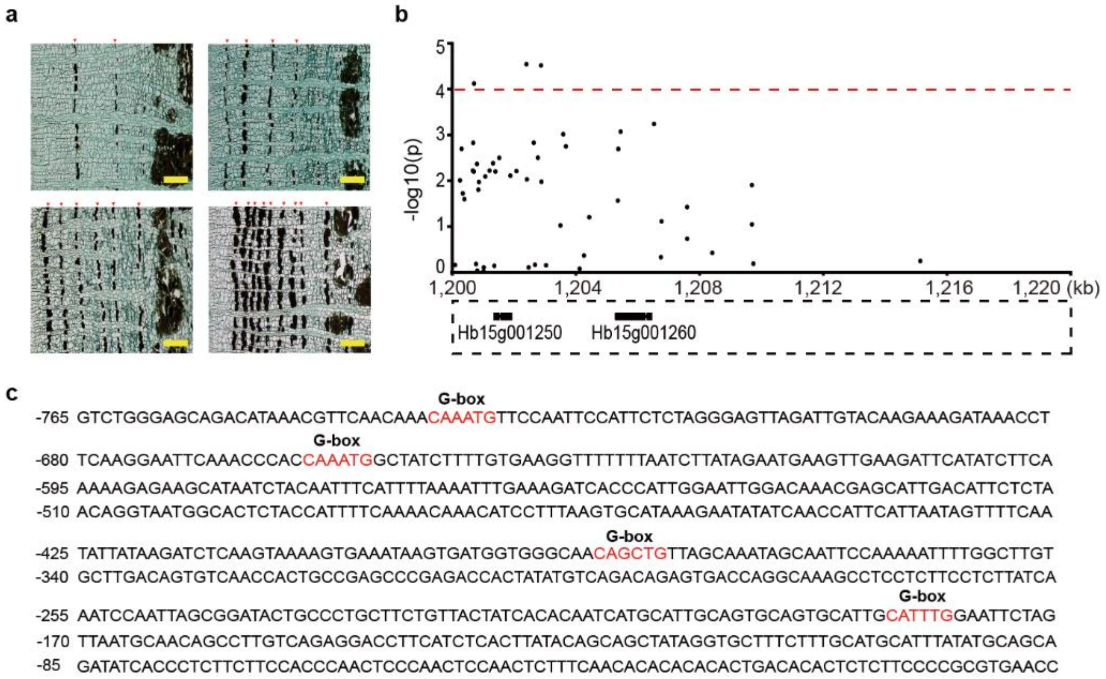


**Supplementary Figure 6. Evaluation of number of laticifer rings and identification of *HbPSK5*.** (a) Light micrographs of bark cross sections showing a difference in the number of laticifer rings among the accessions that were tapped for three years. The red upside-down triangles indicated laticifer rings. Scale bars = 200 μm. (b) The gene structures within the 20-kb overlapped region. (c) G-box located in the promoter region of *HbPSK5*. *Cis*-acting elements were detected by PlantCare analysis.

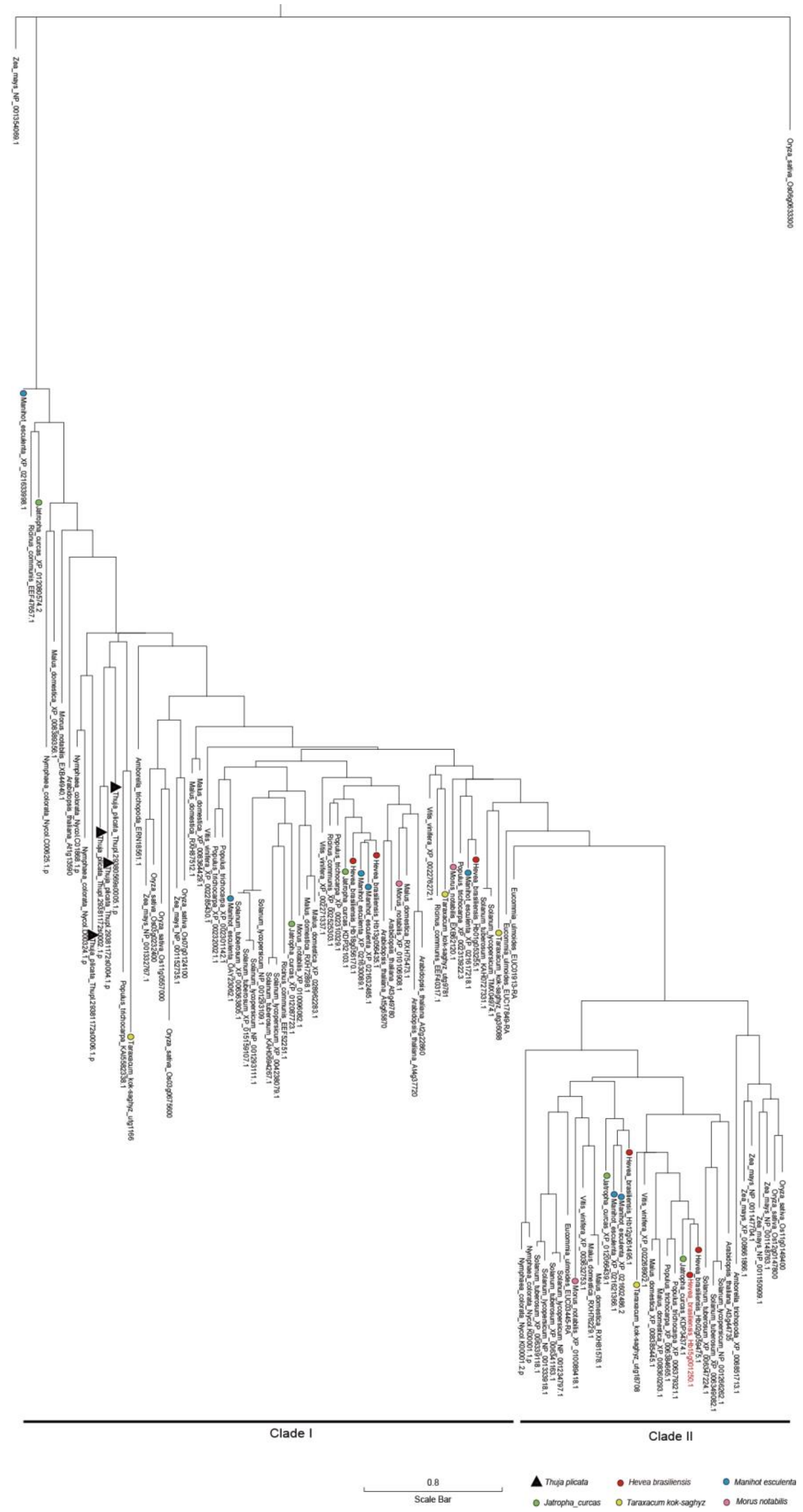


**Supplementary Figure 7. Phylogenetic analysis of PSK proteins from 18 species.** Black triangle marked PSK members from *Thuja plicata* (Gymnospermae). Colored circles indicated PSK members from laticifer-contained plants. HbPSK5 was marked by red font.

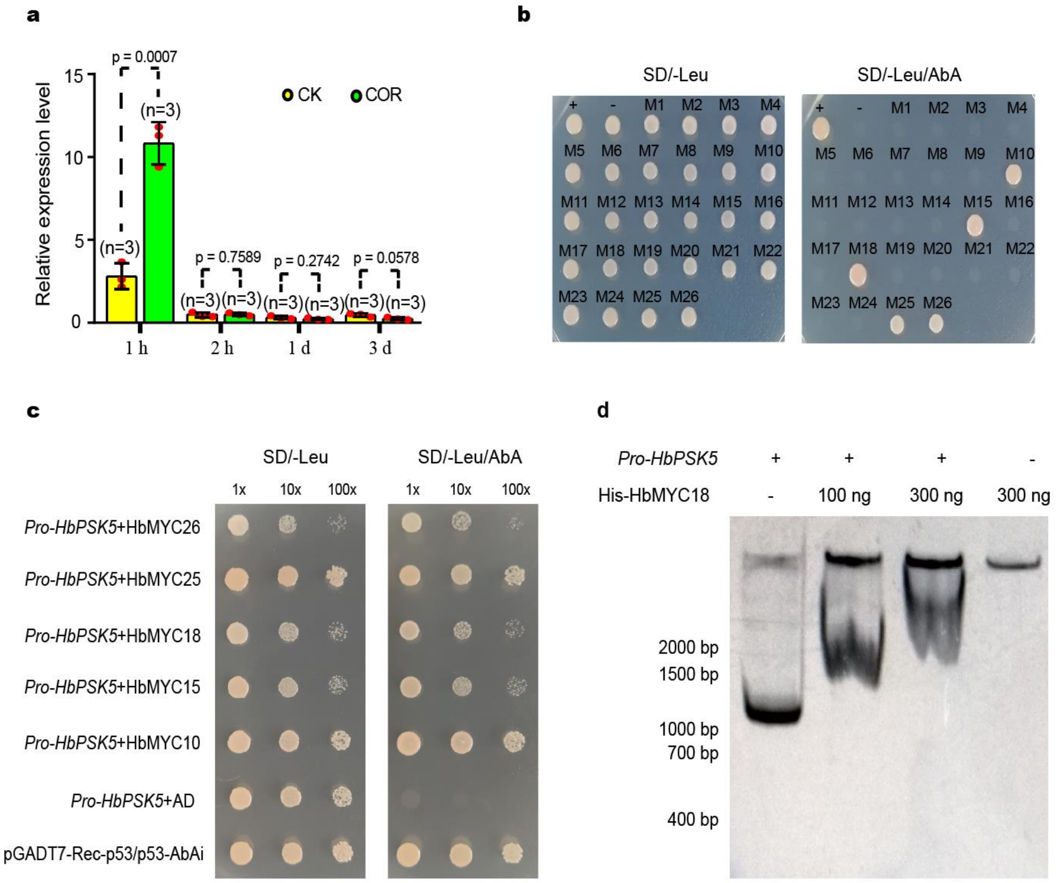


**Supplementary Figure 8. Interaction of HbMYCs and *HbPSK5* promoter by yeast one-hybrid and EMSA assay.** (a) The expression pattern of *HbPSK5* in the cambial region of control (CK) or COR-treated (COR) epicormic shoots. The results are expressed as means ± s.d. in three biological replicates. *P* values were calculated with two-sided Student's *t*-test. ***$P < 0.001$. (b) Coexpression of 26 *HbMYC* members with the *HbPSK5* promoter. (c) Validation of the five positive HbMYCs by yeast one-hybrid. (d) Verification of the physical binding of the HbMYC18 protein to the promoter of *HbPSK5* by EMSA in two replicates. Source data are provided as a Source Data file.

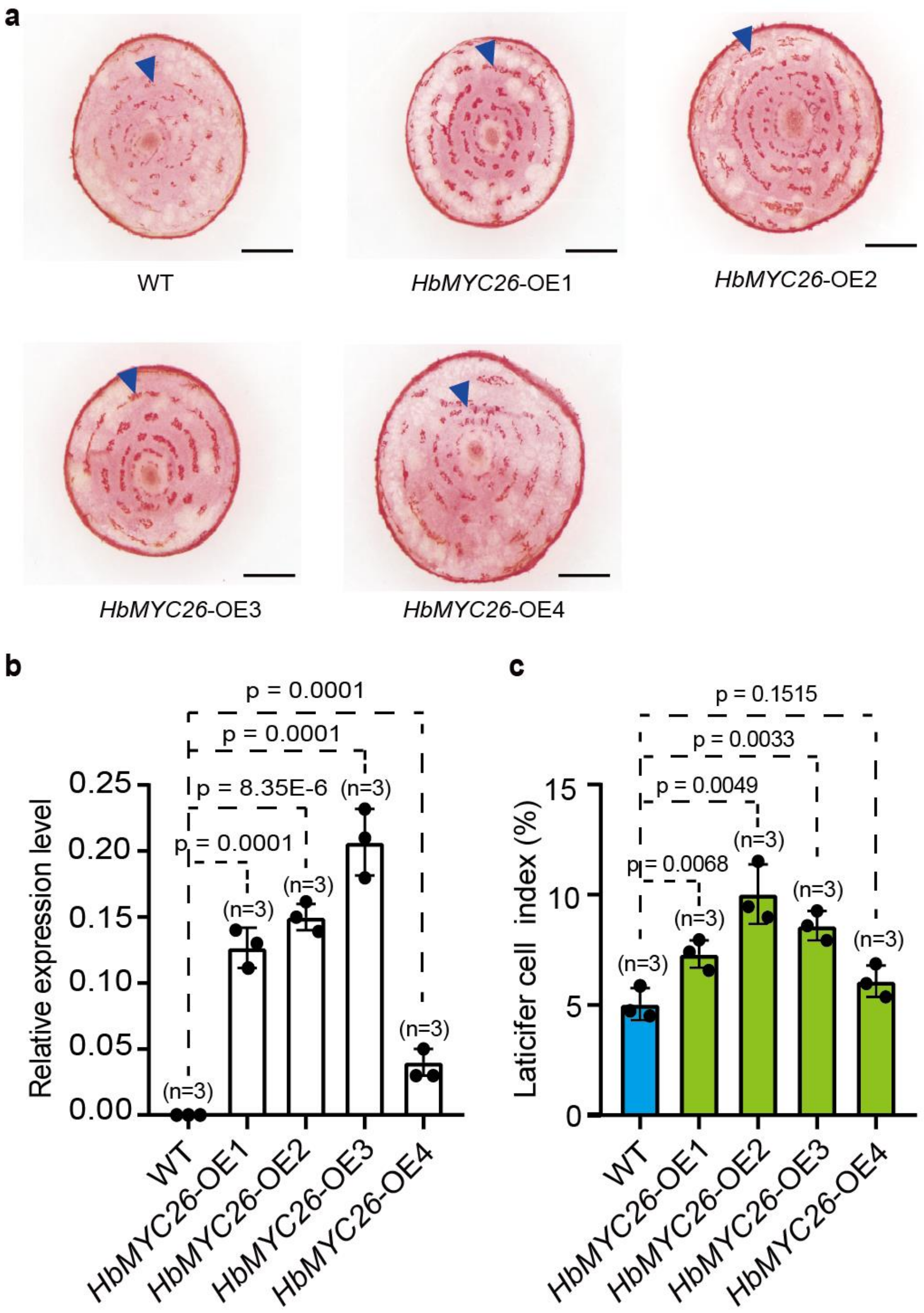


**Supplementary Figure 9. Functional identification of *HbMYC26* in TKS.** (a) Representative light micrographs of root cross sections of WT and *HbMYC26*-overexpressed lines in three biological replicates. The laticifer cells (blue arrowheads) were colored in deep red. Scale bars = 1 mm. (b) Expression levels of *HbMYC26* in WT and *HbMYC26*-overexpressed lines. The results are expressed as means ± s.d. in three biological replicates. *P* values were calculated with two-sided Student's *t*-test. $^{**}P < 0.01$; $^{***}P < 0.001$; $^{****}P < 0.0001$. (c) Laticifer cell index in the root of WT and *HbMYC26*-overexpressed TKS lines. The results are expressed as means ± s.d. in three biological replicates. *P* values were calculated with two-sided Student's *t*-test. $^{**}P < 0.01$. Source data are provided as a Source Data file.

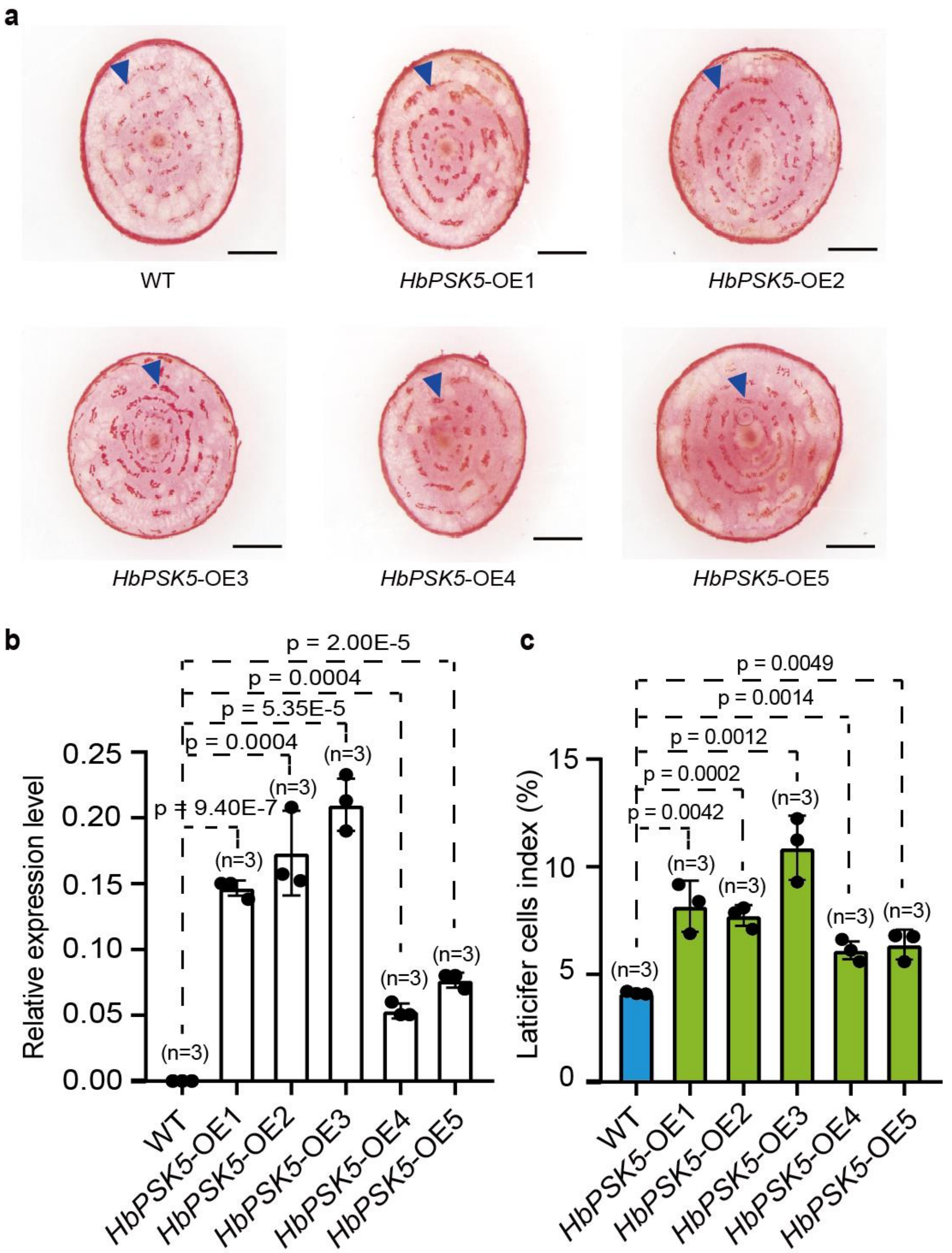


**Supplementary Figure 10. Functional identification of *HbPSK5* in TKS.** (a) Representative light micrographs of root cross sections of WT and *HbPSK5*-overexpressed lines in three biological replicates. The laticifer cells (blue arrowheads) were colored in deep red. Scale bars = 1 mm. (b) Expression levels of *HbPSK5*in WT and *HbMYC26*-overexpressed lines. The results are expressed as means ± s.d. in three biological replicates. *P* values were calculated with two-sided Student's *t*-test. ***P* < 0.01; ****P* < 0.001; *****P* < 0.0001. (c) Laticifer cell index in the root of WT and *HbPSK5*-overexpressed TKS lines. The results are expressed as means ± s.d. in three biological replicates. *P* values were calculated with two-sided Student's *t*-test. ***P* < 0.01. Source data are provided as a Source Data file.

**Supplementary Table 1. Size estimation of rubber tree clone CATAS8-79 genome by the *k*-mer distribution.**

| Total sequence read number | The average length of sequence read (bp) | *k*-mer length | Total number of low frequency *k*-mer | The overall depth estimated from *k*-mer distribution | Genome size |
|---|---|---|---|---|---|
| 570,529,388 | 150 | 17 | 1,282,885,189 | 50 | 1,503,361,055 |
| 570,529,388 | 150 | 19 | 1,945,729,055 | 47 | 1,503,361,056 |
| 570,529,388 | 150 | 21 | 2,265,914,525 | 45 | 1,597,842,354 |
| 570,529,388 | 150 | 23 | 2,487,322,738 | 44 | 1,603,191,794 |
| 570,529,388 | 150 | 25 | 2,670,043,644 | 43 | 1,609,689,705 |
| 570,529,388 | 150 | 27 | 2,820,662,545 | 42 | 1,617,261,466 |

**Supplementary Table 2. BioNano information for CATAS8-79 assembly.**

| Feature of optical maps | |
|---|---|
| **Basic data generated by BspQI enzyme digestion** | |
| Quantity (Gbp) | 206.85 |
| Avg. N50 (Kbp) (>=150kb) | 300.8 |
| Avg. N50 (Kbp) (>=20kb) | 264.7 |
| Avg. label density (per 100 Kbp) | 8.52 |
| Avg. map rate (%) | 53.70% |
| **Optical map assembly** | |
| Number genome maps | 1109 |
| Total genome map length (Mbp) | 2701.09 |
| Mean genome map length (Mbp) | 2.436 |
| Median genome map length (Mbp) | 1.532 |
| Genome map N50 (Mbp) | 3.801 |
| Total reference length (Mbp) | 2088.074 |
| Total genome map length/Reference length | 1.294 |
| Total number of aligned genome maps | 1098 (0.99) |
| Total aligned length (Mbp) | 2320.746 |
| Total aligned length/Reference length | 1.111 |
| Total unique aligned length (Mbp) | 1650.289 |
| Total unique aligned length/Reference length | 0.79 |

**Supplementary Table 3. Statistics for assembly of CATAS8-79 genome.**

| Chromosome | Gap number | Length (bp) |
|---|---|---|
| Chr1 | 35 | 103,215,128 |
| Chr2 | 6 | 94,815,857 |
| Chr3 | 16 | 112,078,056 |
| Chr4 | 22 | 85,260,876 |
| Chr5 | 6 | 105,373,989 |
| Chr6 | 10 | 92,799,857 |
| Chr7 | 13 | 64,494,429 |
| Chr8 | 15 | 70,631,392 |
| Chr9 | 3 | 63,859,080 |
| Chr10 | 14 | 52,797,518 |
| Chr11 | 11 | 72,102,290 |
| Chr12 | 21 | 100,268,846 |
| Chr13 | 9 | 94,713,098 |
| Chr14 | 12 | 52,492,978 |
| Chr15 | 22 | 98,271,093 |
| Chr16 | 8 | 112,327,953 |
| Chr17 | 34 | 82,893,281 |
| Chr18 | 25 | 95,910,900 |
| Total | 282 | 1,554,306,621 |

**Supplementary Table 4. Annotation of repeats in CATAS8-79 assembly.**

| | Length (bp) | Percentage of genome |
|---|---|---|
| **Class I:Retrotransposon** | 946,288,831 | 60.88% |
| LTR-Retrotransposon | 929,105,147 | 59.77% |
| LTR/Copia | 229,070,420 | 14.74% |
| LTR/Gypsy | 686,195,227 | 44.14% |
| LTR-other | 13,839,500 | 0.89% |
| Non-LTR retrotransposon | 17,183,684 | 1.11% |
| SINE | 78,335 | 0.01% |
| LINE | 17,105,349 | 1.10% |
| **Class II:DNA Transposon** | 11,094,572 | 0.71% |
| EnSpm/CACTA | 2,804,304 | 0.18% |
| Harbinger | 374,682 | 0.02% |
| Helitron | 2,083,689 | 0.13% |
| MuDR | 2,412,732 | 0.16% |
| Tcl/Mariner | 285,790 | 0.02% |
| hAT | 2,609,166 | 0.17% |
| DNA-other | 524,209 | 0.03% |
| **Low complexity** | 5,565,222 | 0.36% |
| **Tandem repeat** | 42,508,572 | 2.73% |
| **Unclassified** | 174,661,477 | 11.24% |
| **Total content** | 1,180,118,674 | 75.92% |

**Supplementary Table 5. Genome annotation of allset and coreset.**

| Feature | Allset | Coreset |
|---|---|---|
| Gene count | 102,356 | 38,595 |
| Gene max (bp) | 273,469 | 75,969 |
| Gene min (bp) | 102 | 102 |
| Gene Median/AVG (bp) | 4,381/5,599.87 | 3,680/5,282.54 |
| mRNA count | 102,428 | 82,641 |
| mRNA max (bp) | 21,964 | 21,964 |
| mRNA min (bp) | 102 | 102 |
| mRNA median/AVG (bp) | 1,047/1,405.43 | 1,396/1,680.61 |
| mRNA count median/AVG | 1/1.00 | 1/1.00 |
| CDS median/AVG (bp) | 981/1323.25 | 1,023/1,275.52 |
| Protein median/AVG (aa) | 327/441.08 | 341/425.17 |
| Exon median/AVG (bp) | 110/187.89 | 130/217.09 |
| Exon count median/AVG | 6/7.47 | 6/7.74 |
| UTR3 median/AVG (bp) | 269/406.23 | 137/211.01 |
| UTR5 median/AVG (bp) | 73/179.26 | 95/154.07 |
| Intron median/AVG (bp) | 281/610.86 | 201/552.46 |
| Intron count median/AVG | 5/6.47 | 5/6.74 |

**Supplementary Table 6. Enriched KEGG categories (*q* value < 0.05) expanded and contracted gene families in rubber tree.**

| ID | Description | *q* value |
|---|---|---|
| **Expansion** | | |
| map04016 | MAPK signaling pathway - plant | 6.02E-06 |
| map00900 | Terpenoid backbone biosynthesis | 5.36E-05 |
| map04714 | Thermogenesis | 2.01E-04 |
| map01110 | Biosynthesis of secondary metabolites | 2.91E-04 |
| map05010 | Alzheimer disease | 2.91E-04 |
| map04934 | Cushing syndrome | 7.57E-04 |
| map00960 | Tropane piperidine and pyridine alkaloid biosynthesis | 2.52E-03 |
| map00310 | Lysine degradation | 3.01E-03 |
| map00270 | Cysteine and methionine metabolism | 5.43E-03 |
| map00100 | Steroid biosynthesis | 1.38E-02 |
| map00051 | Fructose and mannose metabolism | 2.72E-02 |
| map03030 | DNA replication | 2.75E-02 |
| map04110 | Cell cycle | 2.75E-02 |
| map00330 | Arginine and proline metabolism | 2.75E-02 |
| map00071 | Fatty acid degradation | 2.76E-02 |
| map00511 | Other glycan degradation | 3.00E-02 |
| map00380 | Tryptophan metabolism | 3.00E-02 |
| **Contraction** | | |
| map03010 | Ribosome | 7.18E-14 |
| map00140 | Steroid hormone biosynthesis | 1.20E-04 |
| map00480 | Glutathione metabolism | 2.02E-04 |
| map00040 | Pentose and glucuronate interconversions | 1.59E-03 |
| map04013 | MAPK signaling pathway - fly | 1.58E-02 |
| map04130 | SNARE interactions in vesicular transport | 1.58E-02 |
| map05034 | Alcoholism | 1.68E-02 |

**Supplementary Table 7. Information of SNP and InDel.**

| Category | SNP | InDel |
|---|---|---|
| Total | 5,323,701 | 259,445 |
| Insertion | - | 78,989 |
| Deletion | - | 180,456 |
| Intergenic | 3,348,782 | 137,735 |
| Upstream | 194,674 | 16,072 |
| Downstream | 191,077 | 15,332 |
| Upstream/Downstream | 20,065 | 1,903 |
| UTR3 | 35,618 | 3,497 |
| UTR5 | 14,801 | 1,418 |
| UTR5/UTR3 | 100 | 8 |
| Intronic | 1,056,475 | 72,806 |
| Splicing | 5,964 | 368 |
| Exonic | 456,096 | 10,304 |
| Exonic;Splicing | 49 | 2 |
| Stop gain | 9,645 | 181 |
| Stop loss | 692 | 19 |
| Frameshift insertion | - | 1,858 |
| Frameshift deletion | - | 4,922 |
| Nonframeshift insertion | - | 592 |
| Nonframeshift deletion | - | 2,150 |
| Synonymous | 173,149 | - |
| Non-synonymous | 244,969 | - |
| Unknowns | 27,690 | 584 |

**Supplementary Table 8. *F*st values between groups.**

| Groups | WAC | WRO1 | WRO2 | WMG |
|---|---|---|---|---|
| WAC | | | | |
| WRO1 | 0.142 | | | |
| WRO2 | 0.193 | 0.089 | | |
| WMG | 0.393 | 0.338 | 0.349 | |
| Cul | 0.459 | 0.388 | 0.395 | 0.138 |

**Supplementary Table 9. Tissue expression patterns of six PSK genes in rubber tree.**

| **Gene name** | **FPKM (different tissues)** | | | | | | |
|---|---|---|---|---|---|---|---|
| | **Cambium region** | **Inner bark** | **Virgin latex** | **Tapping latex** | **Female flower** | **Male flower** | **Leaves** |
| *HbPSK1* (Hb01g053255) | 0.00 | 7.93 | 0.00 | 0.00 | 2.45 | 10.20 | 0.00 |
| *HbPSK2* (Hb02g059475) | 0.00 | 6.94 | 0.00 | 0.00 | 1.11 | 10.17 | 2.26 |
| *HbPSK3* (Hb12g061495) | 84.57 | 8.51 | 1996.31 | 333.35 | 127.31 | 84.59 | 3.69 |
| *HbPSK4* (Hb13g056435) | 15.29 | 0.00 | 65.78 | 39.60 | 4.69 | 11.91 | 100.67 |
| *HbPSK5* (Hb15g001250) | 1.01 | 0.47 | 0.00 | 0.00 | 0.00 | 0.00 | 0.00 |
| *HbPSK6* (Hb18g056170) | 308.31 | 6.02 | 0.81 | 2.41 | 91.60 | 113.30 | 0.43 |
