# Supplementary material for: Genomic insight into domestication of rubber tree
Source: Nat Commun. 2023 Aug 2;14:4651. doi: 10.1038/s41467-023-40304-y (PMC10397287; doi:10.1038/s41467-023-40304-y)
Supplement: Supplementary file 3 — Description of Additional Supplementary Files [file 41467_2023_40304_MOESM3_ESM.pdf]

Description of Additional Supplementary Files
File Name: Supplementary Data 1
Description: Global statistical comparison between CATAS8-79 assembly and the assembly of available rubber tree clones.

File Name: Supplementary Data 2
Description: Paired-end transcriptome sequencing data for seven tissues of CATAS8-79.

File Name: Supplementary Data 3
Description: Summary of gene families in 12 species.

File Name: Supplementary Data 4
Description: Expression patterns of genes related to natural rubber biosynthesis, JA and ETH signaling pathways in seven tissues.

File Name: Supplementary Data 5
Description: Amino acid sequences of MYC proteins in six species.

File Name: Supplementary Data 6
Description: Re-sequenced information of rubber tree accessions.

File Name: Supplementary Data 7
Description: The number of laticifer ring (NLR) and latex production of 208 cultivated and wild germplasms.

File Name: Supplementary Data 8
Description: List of selected genes in the selective sweeps by Fst method.

File Name: Supplementary Data 9
Description: List of selected genes in the selective sweeps by πmethod.

File Name: Supplementary Data 10
Description: List of selected genes in the selective sweeps by XP-CLR method.

File Name: Supplementary Data 11
Description: Haplotypes in rubber productivity-related genes.

File Name: Supplementary Data 12
Description: Tissue-specific expression of 32 putative genes associated with NLR.

File Name: Supplementary Data 13
Description: The SNP Chr15:1200714 validation.

File Name: Supplementary Data 14
Description: Distribution of three genotypes in 335 accessions.

File Name: Supplementary Data 15
Description: Distribution of three genotypes in the individuals with more laticifer rings (ML) and fewer

laticifer rings (FL) from hybrid segregation populations.

File Name: Supplementary Data 16
Description: Number of PSK genes plant taxa.

File Name: Supplementary Data 17
Description: Amino acid sequences of PSK proteins in plants.

File Name: Supplementary Data 18
Description: Primers used in this study.
