# Supplementary material for: Genomic insight into domestication of rubber tree
Source: Nat Commun. 2023 Aug 2;14:4651. doi: 10.1038/s41467-023-40304-y (PMC10397287; doi:10.1038/s41467-023-40304-y)
Supplement: Supplementary file 22 — Reporting Summary [file 41467_2023_40304_MOESM22_ESM.pdf]

nature portfolio

Corresponding author(s): Jiayang Li, Chengzhi Liang, & Wei-Min Tian

Last updated by author(s): Jul 13, 2023

Reporting Summary

Nature Portfolio wishes to improve the reproducibility of the work that we publish. This form provides structure for consistency and transparency in reporting. For further information on Nature Portfolio policies, see our Editorial Policies and the Editorial Policy Checklist.

Statistics

For all statistical analyses, confirm that the following items are present in the figure legend, table legend, main text, or Methods section.

n/a	Confirmed	
☐	☒	The exact sample size (*n*) for each experimental group/condition, given as a discrete number and unit of measurement
☐	☒	A statement on whether measurements were taken from distinct samples or whether the same sample was measured repeatedly
☐	☒	The statistical test(s) used AND whether they are one- or two-sided<br>*Only common tests should be described solely by name; describe more complex techniques in the Methods section.*
☒	☐	A description of all covariates tested
☐	☒	A description of any assumptions or corrections, such as tests of normality and adjustment for multiple comparisons
☐	☒	A full description of the statistical parameters including central tendency (e.g. means) or other basic estimates (e.g. regression coefficient) AND variation (e.g. standard deviation) or associated estimates of uncertainty (e.g. confidence intervals)
☐	☒	For null hypothesis testing, the test statistic (e.g. F, t, r) with confidence intervals, effect sizes, degrees of freedom and P value noted<br>*Give P values as exact values whenever suitable.*
☒	☐	For Bayesian analysis, information on the choice of priors and Markov chain Monte Carlo settings
☒	☐	For hierarchical and complex designs, identification of the appropriate level for tests and full reporting of outcomes
☒	☐	Estimates of effect sizes (e.g. Cohen's *d*, Pearson's *r*), indicating how they were calculated

Our web collection on statistics for biologists contains articles on many of the points above.

Software and code

Policy information about availability of computer code

Data collection	No software was used in data collection.
Data analysis	Jellyfish (version 2.2.3); SMRTAnalysis package (version 3.1); CANU (version 1.7); IrysSolve (version 3.0); Pilon (version 1.22); BUSCO (version 4.1.4); RepeatMasker (version 4.0.6); RepeatMasker (version 4.0.6); LTR_FINDER (version 1.06); Trinity (version 2.4.0); AUGUSTUS (version 3.2.3), GeneScan (version 1.0), GlimmerHMM (version 3.04), SNAP (https://github.com/KorfLab/SNAP); EVidenceModeler (version 1.1.1); Orthofinder (version 2.3.3); MUSCLE (version 3.8.31); RAxML (version 8.2.12); PAML (version 4.5); BWA (version 0.7.17-r1188); SAMtools (version 1.3); GATK (version 3.4); snpEff (version 4.3); fastStructure (version 1.0); SNPRelate (version 1.16.0); VCFtools (version 0.1.15); Plink (version 1.90b5); XP-CLR (version 1.0)

For manuscripts utilizing custom algorithms or software that are central to the research but not yet described in published literature, software must be made available to editors and reviewers. We strongly encourage code deposition in a community repository (e.g. GitHub). See the Nature Portfolio guidelines for submitting code & software for further information.

Data

Policy information about availability of data

All manuscripts must include a data availability statement. This statement should provide the following information, where applicable:

- Accession codes, unique identifiers, or web links for publicly available datasets
- A description of any restrictions on data availability
- For clinical datasets or third party data, please ensure that the statement adheres to our policy

The genome assembly and annotation generated in this study have been uploaded to the open dissemination research data repository Zenodo with a website: https://doi.org/10.5281/zenodo.7123623. The raw sequencing data and transcriptome sequencing data generated in this study have been deposited to the National Genomics Data Center (NGDC, https://ngdc.cncb.ac.cn/) under project number PRJCA004986. Published transcriptome sequencing data (Gene Expression Omnibus (GEO) accession number GSE80596 [https://www.ncbi.nlm.nih.gov/gds/?term=GSE80596]) is used for expression analysis in the present study.

Human research participants

Policy information about studies involving human research participants and Sex and Gender in Research.

Reporting on sex and gender	This work does not involve human research.
Population characteristics	N/A
Recruitment	N/A
Ethics oversight	N/A

Note that full information on the approval of the study protocol must also be provided in the manuscript.

Field-specific reporting

Please select the one below that is the best fit for your research. If you are not sure, read the appropriate sections before making your selection.

☒ Life sciences ☐ Behavioural & social sciences ☐ Ecological, evolutionary & environmental sciences

For a reference copy of the document with all sections, see nature.com/documents/nr-reporting-summary-flat.pdf

Life sciences study design

All studies must disclose on these points even when the disclosure is negative.

Sample size	127 cultivars and 208 wild accessions were selected from the National Tropical Plants Germplasm Resource Center-Rubber Tree, enoughly representing the cultivated and wild accessions to query meaningful and decisive conclusion.
Data exclusions	For genome and RNA-Seq data, we excluded sequences that were of low quality and potential contaminants. This is standard for such analyses. For population genomic analysis, we excluded accessions that were clustered into unexpected groups based on the phylogeny analysis.
Replication	For qRT-PCR experiments and the measurement of NLR and latex production, we used three biological replicates. For EMSA and semi-quantitative PCR, we used two biological replicates. For morphological experiments, we used five biological replicates. For transient dual-luciferase assays, we used 16 biological replicates. For genetic transformation, we used five biological replicates.
Randomization	This is not relevant to our study.
Blinding	Blinding was not relevant to our study.

Reporting for specific materials, systems and methods

We require information from authors about some types of materials, experimental systems and methods used in many studies. Here, indicate whether each material, system or method listed is relevant to your study. If you are not sure if a list item applies to your research, read the appropriate section before selecting a response.

Materials & experimental systems

n/a	Involved in the study
x	Antibodies
x	Eukaryotic cell lines
x	Palaeontology and archaeology
x	Animals and other organisms
x	Clinical data
x	Dual use research of concern

Methods

n/a	Involved in the study
x	ChIP-seq
x	Flow cytometry
x	MRI-based neuroimaging
